# Effects of Cholecalciferol on Key Components of Vitamin D-Endo/Para/Autocrine System in Experimental Type 1 Diabetes

**DOI:** 10.1155/2018/2494016

**Published:** 2018-02-06

**Authors:** Anna Mazanova, Ihor Shymanskyi, Olha Lisakovska, Lala Hajiyeva, Yulia Komisarenko, Mykola Veliky

**Affiliations:** ^1^Department of Biochemistry of Vitamins and Coenzymes, Palladin Institute of Biochemistry of the National Academy of Sciences of Ukraine, Kyiv, Ukraine; ^2^Department of Endocrinology, Bogomolets National Medical University, Kyiv, Ukraine

## Abstract

**Objectives:**

Recent prospective studies have found the associations between type 1 diabetes (T1D) and vitamin D deficiency. We investigated the role of vitamin D in the regulation of 25OHD-1*α*-hydroxylase (CYP27B1) and VDR expression in different tissues of T1D rats.

**Design:**

T1D was induced in male Wistar rats by streptozotocin (55 mg/k b.w.). After 2 weeks of T1D, the animals were treated orally with or without vitamin D_3_ (cholecalciferol; 100 IU/rat, 30 days).

**Methods:**

Serum 25-hydroxyvitamin D (25OHD) was detected by ELISA. CYP27A1, CYP2R1, CYP27B1, and VDR were assayed by RT-qPCR and Western blotting or visualized by immunofluorescence staining.

**Results:**

We demonstrated that T1D led to a decrease in blood 25OHD, which is probably due to the established downregulation of CYP27A1 and CYP2R1 expression. Vitamin D deficiency was accompanied by elevated synthesis of renal CYP27B1 and VDR. Conversely, CYP27B1 and VDR expression decreased in the liver, bone tissue, and bone marrow. Cholecalciferol administration countered the impairments of the vitamin D-endo/para/autocrine system in the kidneys and extrarenal tissues of diabetic rats.

**Conclusions:**

T1D-induced vitamin D deficiency is associated with impairments of renal and extrarenal CYP27B1 and VDR expression. Cholecalciferol can be effective in the amelioration of diabetes-associated abnormalities in the vitamin D-endo/para/autocrine system.

## 1. Introduction

Vitamins of D group (predominantly vitamin D_2_ and D_3_) are biologically active substances of secosteroid nature [1]. The effects of vitamin D, which go beyond regulation of calcium homeostasis and bone remodeling, have currently received much attention. Recent evidence suggests that hormonally active form of vitamin D, 1,25(OH)_2_D (calcitriol), is able to regulate important cellular processes such as proliferation and differentiation, immune responses, osteoimmunity, angiogenesis, and apoptosis [2, 3]. Vitamin D deficiency and disruptions in the vitamin D-endo/para/autocrine system can increase the risk of autoimmune diseases, in particular type 1 diabetes [4]. Several studies have shown that vitamin D plays an important role in preventing pancreatic *β*-cell autoimmune destruction [5]. Calcitriol is reported to be directly involved in insulin production and can improve the sensitivity of peripheral tissues to insulin action [6].

The realization of the biological effects of vitamin D in cells is closely related to the functioning of the vitamin D-endo/para/autocrine system, which includes (1) photoconversion of 7-dehydrocholesterol in the skin with the formation of cholecalciferol; (2) synthesis of 25OHD (calcidiol) in the liver by means of two key vitamin D 25-hydroxylases (сytochromes P450), CYP2R1 and CYP27A1; (3) conversion of 25-hydroxyvitamin D (25OHD) in the kidneys or extrarenal tissues to hormonally active form, 1*α*,25(OH)_2_D, by 25OH-1*α*-hydroxylase (CYP27B1); (4) calcitriol transport to target organs by vitamin D binding protein (VDBP); and (5) binding of 1*α*,25(OH)_2_D to vitamin D receptors (VDR) and regulation of gene expression [7].

While renal activation of CYP27B1 leads to the formation of the vitamin D hormone, 1,25(OH)_2_D, which is mainly responsible for the calcium absorption in the intestine and kidneys, extrarenal calcitriol covers a wider range of noncalcemic physiological functions [2, 3]. There are different cell types that express CYP27B1, such as cutaneous and intestinal epithelial cells, macrophages, and breast and bone cells [8]. The physiological and pharmacological effects of 1,25(OH)_2_D are mediated by VDR, the member of the superfamily of steroid nuclear receptors [2]. Being inherent to a variety of target cells, these receptors provide a significant therapeutic potential of vitamin D as a natural VDR ligand in many diseases.

The relationship between the bioavailability of vitamin D, its metabolism, and T1D development is multifaceted and is confirmed by genetic studies in humans. It has been established that 25OНD deficiency, associated with cytochrome polymorphism and inherited defects in vitamin D metabolism, predisposes to T1D and its complications. In particular, there is a close linkage between the polymorphism of certain alleles of *Cyp2r1* gene, decreased level of circulating 25OHD, and the development of T1D as well as diabetes-associated secondary osteoporosis [9]. The importance of the vitamin D-endo/para/autocrine system is further confirmed by the correlation between T1D development, osteoporosis, and the presence of functionally defective alleles in *Cyp27b1* and *Vdr* genes [10, 11]. Consequently, abnormal activity of vitamin D hydroxylation enzymes and inadequacy of VDR for a correct signal transduction may have relevance to T1D and its complications.

In chronic diabetes, a number of abnormalities are observed in tissues that potentially participate in vitamin D metabolism. However, there is no unambiguous evidence for TD1-associated expression of CYP27B1 and VDR, precisely those components that link vitamin D metabolism and signaling. Therefore, our aim was to investigate how T1D changes tissue distribution of CYP27B1 and VDR and whether vitamin D_3_ (cholecalciferol) treatment can affect diabetes-related dysfunction of the vitamin D-endo/para/autocrine system.

## 2. Materials and Methods

### 2.1. Experimental Design

Male Wistar rats (140 ± 7 g) were injected with streptozotocin at dose 55 mg/kg of b.w. Control animals received 10 mM citrate buffer solution. After two weeks of diabetes induction, the animals were divided into two groups: diabetic rats and diabetic rats, which were treated per os with 100 IU of vitamin D_3_ for 30 days. All animal procedures were performed in accordance with the European Convention for Protection of Vertebrate Animals used for Research and Scientific Purposes (Strasbourg, 1986), General Ethical Principles of Animal Experimentation, approved by the First National Congress on Bioethics (Kyiv, 2001).

### 2.2. Serum 25OHD

The content of 25OHD in the blood serum was determined by the in-house developed ELISA kit [12].

### 2.3. Isolation of Bone Marrow Cells and Immunocytochemistry

The bone marrow cells were obtained according to protocol, which we described previously [13]. For fluorescence visualization, we used primary antibody to CYP27B1 and VDR (1 : 200; Santa Cruz Biotech., USA) and secondary antibodies DyLight 488 and Alexa Fluor 546 (1 : 250; ThermoFisher, USA). Nuclei were stained with Hoechst (0.1 *μ*g/mL; Sigma, USA). Samples were analyzed on a LSM 510 META confocal microscope (100x magnification).

### 2.4. SDS-PAG Electrophoresis and Western Blotting (WB)

Equal protein samples (50 *μ*g) were separated by 10% SDS-PAGE and then transferred onto nitrocellulose membranes. For protein signal detection, we used primary antibodies against VDR, CYP27B1 (1 : 200; Santa Cruz Biotech., USA), *β*-actin (1 : 25000; Sigma, USA), and secondary antibodies: anti-mouse (1 : 2500; Sigma, USA) and anti-goat (1 : 1000; Invitrogen, USA) conjugated with HRP. Membranes were subjected to chemiluminescent detection with p-coumaric acid (Sigma, USA) and luminol (AppliChem GmbH, Germany). The immunoreactive bands were quantified with GelPro Analyzer 3.2.

### 2.5. RNA Extraction and Quantitative Real-Time Polymerase Chain Reaction (RT-qPCR)

Total RNA was extracted with a mini PREP RNA mini kit (Qiagen, USA) according to the manufacturer's protocol. Quantity and purity of the RNA were determined by NanoDrop DeNovix DS-11. Synthesis of cDNA was carried out using a Maxima H Minus First Strand cDNA Synthesis Kit (ThermoFisher, USA) as described by the manufacturer. RT-qPCR was performed with Maxima SYBER Green/ROX qPCR Master Mix (2×) (ThermoFisher, USA) and specific primers against *Vdr* (5′-TCATCCCTACTGTGTCCCGT-3′ sense, 5′-TGAGTGCTCCTTGGTTCGTG-3′ antisense), *Cyp27a1* (5′-TCGACACATCCTGATTGGAAGG-3′ sense, 5′-TCTCATGCGGCTCAACACAG-3′ antisense), *Cyp2r1* (5′-CCTTCTGCTACTACTCGTGC-3′ sense, 5′-GCATGGTCTATCTCGCCAAA-3′ antisense), *Cyp27b1* (5′-TGGGTGCTGGGAACTAACCC-3′ sense, 5′-TCGCAGACTGATTCCACCTC-3′ antisense), and *Gapdh* (5′-TGAACGGGAAGCTCACTGG-3′ sense, 5′-TCCACCACCCTGTTGCTGTA-3′ antisense). Data were calculated using the ΔΔC_t_ method.

### 2.6. Statistical Analysis

All results are expressed as mean ± SEM. The Kolmogorov-Smirnov test was used for testing on normal distribution. Statistical differences between the various groups were compared by using the ANOVA test. Differences were considered significant when *p* ≤ 0.05. All statistical analysis was performed using Origin Pro 8.5 (OriginLab Corporation, Northampton, MA, USA).

## 3. Results

We found that six weeks after the injection of streptozotocin, fasting blood glucose exceeds the control level by 5.5-fold (*p* = 0.0001) (Table 1). Glucose-lowering effect of cholecalciferol was statistically insignificant in diabetes. Chronic hyperglycemia was accompanied by profound changes in blood serum 25OHD content as a marker of vitamin D bioavailability. At week 6, after the initiation of diabetes, serum 25OHD levels were reduced by approximately 50% (*p* = 0.0001) compared with controls (Table 1). Cholecalciferol administration during 30 days significantly improved vitamin D bioavailability.

**Figure 1:**
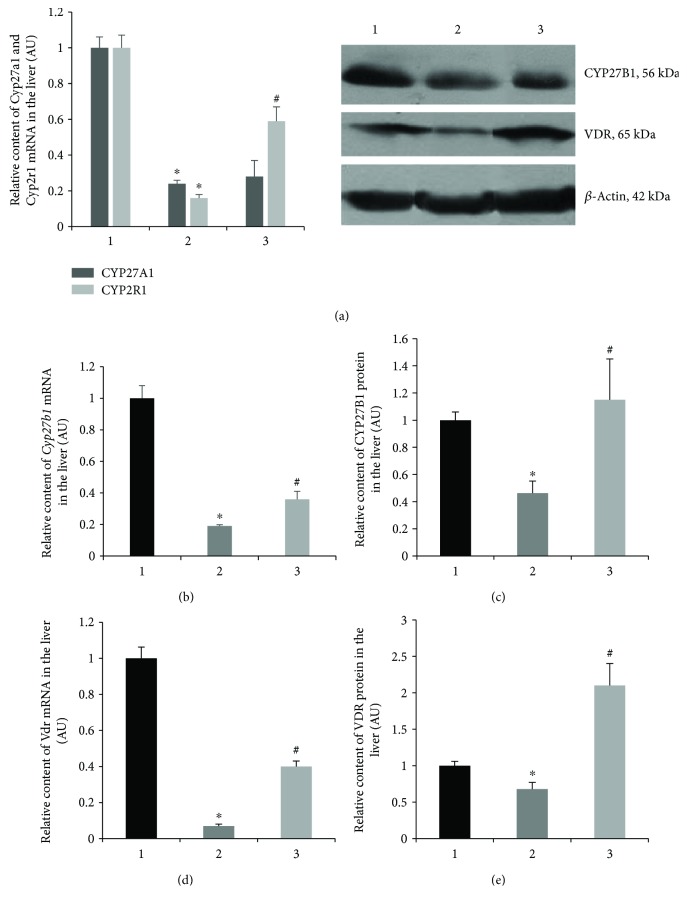
Transcript level (mRNA) of *Cyp27a1*, *Cyp2r1* (a), *Cyp27b1* (b), and *Vdr* (d) and protein abundance of CYP27B1 (c) and VDR (e) in liver tissue of diabetic rats and after vitamin D_3_ treatment. 1—control; 2—diabetic rats; and 3—vitamin D_3_-treated diabetic rats. Findings are shown in representative immunoblots. Results are expressed as mean ± SEM. ^∗^*p* < 0.05 versus control; ^#^*p* < 0.05 versus diabetes, *n* = 6.

Since the conversion of vitamin D to 25OHD predominantly catalyzes two cytochrome P450 isoforms (mitochondrial CYP27A1 and microsomal CYP2R1), it was useful to determine whether a significant 25OHD deficiency in diabetes is associated with any changes in the expression of these enzymes. It was shown that mRNA contents of *Cyp27a1* and *Cyp2r1* in the liver of diabetic rats decreased by 4.2-fold (*p* = 0.039) and 6.3-fold (*p* = 0.033), respectively, as compared with the control (Figure 1(a)). Diabetic rats administered with cholecalciferol demonstrated a 3.7-fold increase in *Cyp2r1* mRNA that corresponds to 60% of the control value (*p* = 0.033). There was no difference in the *Cyp27a1* mRNA expression after vitamin D_3_ treatment compared with the diabetes group.

Next, we investigated the distribution of vitamin D-endo/para/autocrine system components, such as CYP27B1 and VDR in different tissues. Their mRNA and protein levels were determined in classical (metabolic) tissues (liver and kidneys) and nonclassical tissues (bone and bone marrow). According to RT-qPCR data, the *Cyp27b1* mRNA level in the liver of diabetic animals was significantly reduced (by 5.3-fold) as compared with the control (*p* = 0.035) (Figure 1(b)). Downregulation of cytochrome was further confirmed by WB (Figure 1(c)). Diabetic animals exhibited the CYP27B1 protein level 2.2-fold lower than in the control (*p* = 0.001). As shown in Figures 1(d) and 1(e), similar changes in the expression of VDR at the transcriptional and translational levels were seen. The *Vdr* mRNA level was detected to be 14.3-fold less pronounced than in the control (*p* = 0.0004). As evidenced by the WB data, the VDR protein level in diabetes decreased 1.5-fold in comparison with the control (*p* = 0.009). Cholecalciferol treatment resulted in a 2.0-fold (*p* = 0.035) and 5.7-fold (*p* = 0.0004) elevation of *Cyp27b1* and *Vdr* mRNAs, respectively, compared with diabetic animals. While fully restoring the protein level of CYP27B1, cholecalciferol caused an increase in the protein VDR level that reached the value 2.2-fold higher compared with the control (*p* = 0.009).

**Figure 2:**
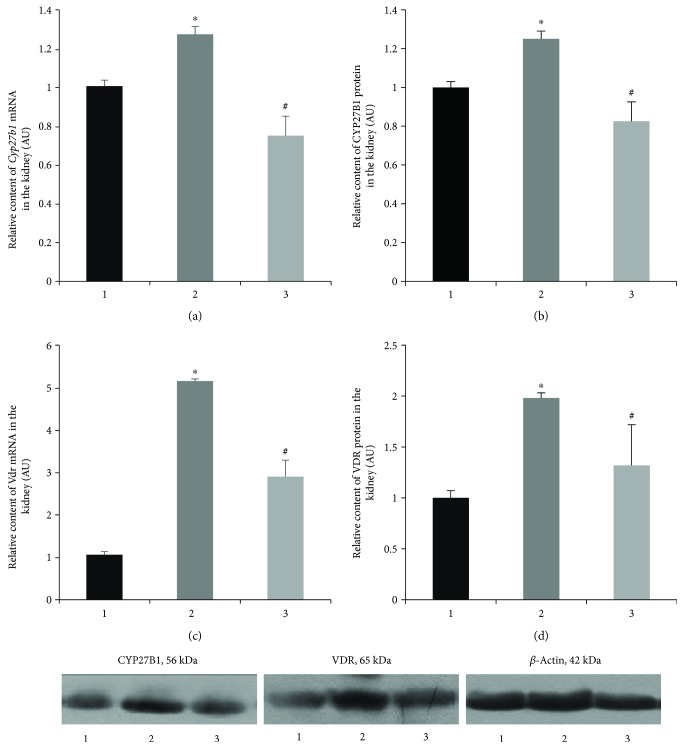
Transcript level (mRNA) and protein abundance of CYP27B1 (a, b) and VDR (c, d) in kidney tissue of diabetic rats and after vitamin D_3_ treatment. 1—control; 2—diabetic rats; and 3—vitamin D_3_-treated diabetic rats. Findings are shown in representative immunoblots. Results are expressed as mean ± SEM. ^∗^*p* < 0.05 versus control; ^#^*p* < 0.05 versus diabetes, *n* = 6.

Conversely, in the kidney tissue, there were opposite changes in the vitamin D-endocrine system. Vitamin D-deficient status of the animals with T1D leads to an increase in CYP27B1 at both transcriptional (by 1.28-fold, *p* = 0.003) and translational (by 1.25-fold, *p* = 0.005) levels in the renal tissue (Figures 2(a) and 2(b)). The same tendency, but more pronounced, was observed with respect to mRNA and protein content of VDR (Figures 2(c) and 2(d)). When measured by RT-qPCR, the level of *Vdr* mRNA in diabetic animals was 5.2-fold higher than that in the control (*p* = 0.048), which was accompanied by a 2.0-fold increase in the VDR protein level (*p* = 0.0002). As shown in Figure 2, the administration of cholecalciferol partially or completely abrogated the effects of diabetes on CYP27B1 and VDR expression in the kidneys.

**Figure 3:**
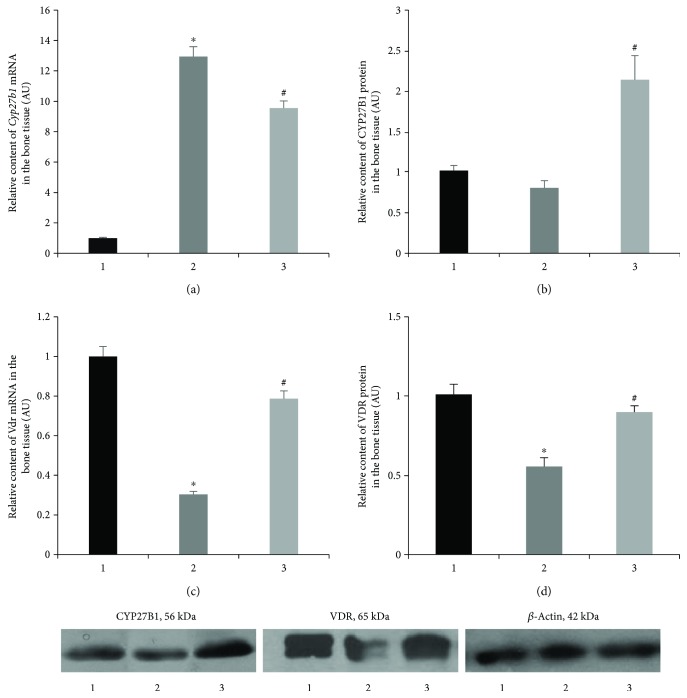
Transcript level (mRNA) and protein abundance of CYP27B1 (a, b) and VDR (c, d) in bone tissue of diabetic rats and after vitamin D_3_ treatment. 1—control; 2—diabetic rats; and 3—vitamin D_3_-treated diabetic rats. Findings are shown in representative immunoblots. Results are expressed as mean ± SEM. ^∗^*p* < 0.05 versus control; ^#^*p* < 0.05 versus diabetes, *n* = 6.

It is known that the bones possess one of the most active vitamin D-para/autocrine systems. As shown in Figure 3(a), T1D caused a significant 13.0-fold elevation of *Cyp27b1* mRNA in bone tissue (*p* = 0.006). Unexpectedly, WB revealed no significant changes in the synthesis of CYP27B1 protein (Figure 3(b)). Opposite results were observed for VDR expression in the bone tissue (Figures 3(c) and 3(d)), where a significant 3.3-fold reduction in *Vdr* mRNA was detected (*p* = 0.0003). Consistent with the diabetes-induced lowering of *Vdr* expression, there was a 1.8-fold decrease in the protein level of VDR in the bones (*p* = 0.0001). With a significant normalizing effect on *Cyp27b1* gene transcription, cholecalciferol treatment increased CYP27B1 protein to a level twice that of the control. Vitamin D_3_ partially or completely corrected VDR expression in the bone tissue.

**Figure 4:**
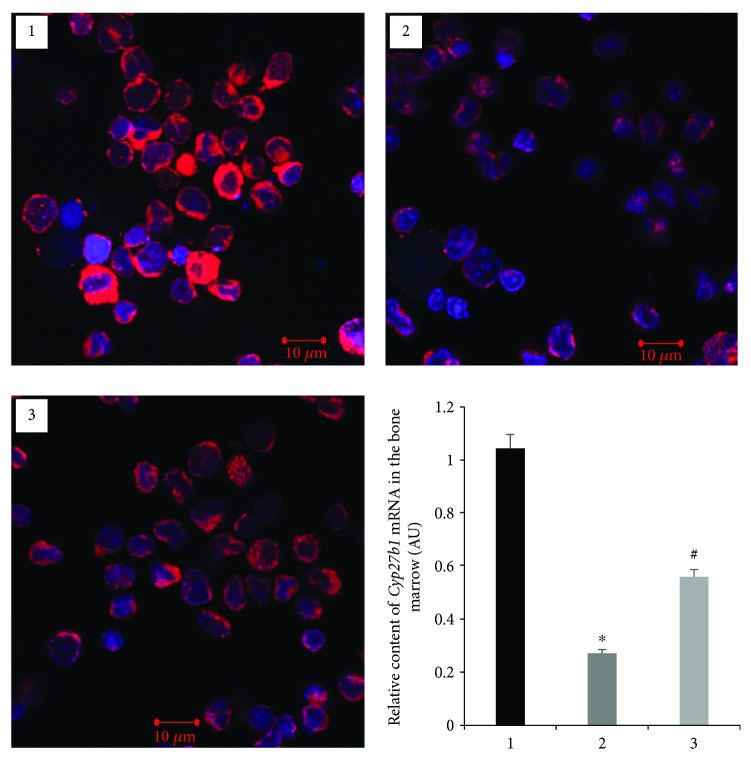
Immunofluorescence staining and transcript level (mRNA) of *Cyp27b1* in the bone marrow of diabetic rats and after vitamin D_3_ treatment. 1—control; 2—diabetic rats; and 3—vitamin D_3_-treated diabetic rats. Harvested bone marrow cells were stained for CYP27B1 (red fluorescence; scale bars, 10 *μ*m). Nuclei were counterstained with Hoechst. Results of RT-qPCR are expressed as mean ± SEM. ^∗^*p* < 0.05 versus control; ^#^*p* < 0.05 versus diabetes, *n* = 6.

**Figure 5:**
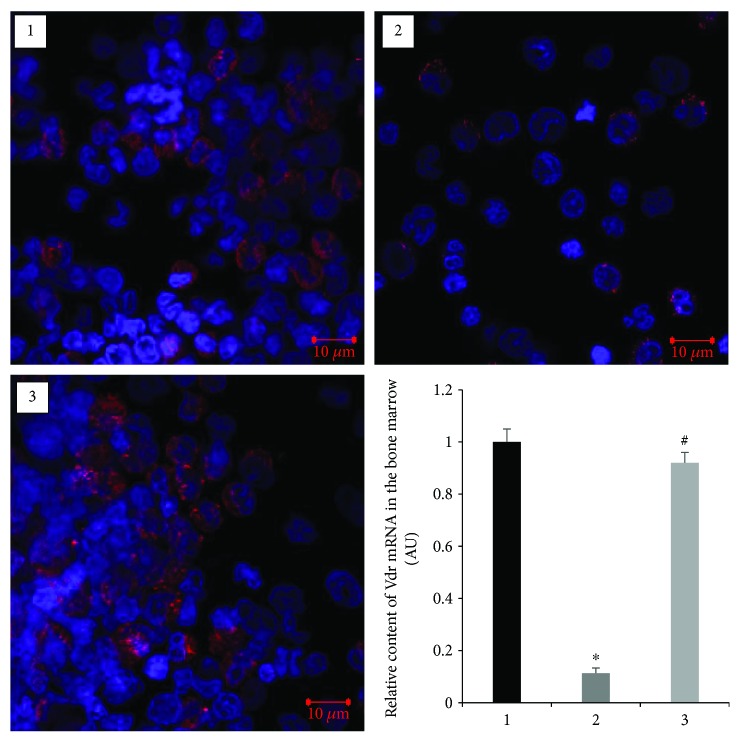
Immunofluorescence staining and transcript level (mRNA) of VDR in the bone marrow of diabetic rats and after vitamin D_3_ treatment. 1—control; 2—diabetic rats; and 3—vitamin D_3_-treated diabetic rats. Harvested bone marrow cells were stained for VDR (red fluorescence; scale bars, 10 *μ*m). Nuclei were counterstained with Hoechst. RT-qPCR data are expressed as mean ± SEM. ^∗^*p* < 0.05 versus control; ^#^*p* < 0.05 versus diabetes, *n* = 6.

To further determine that altered CYP27B1 and VDR expression occurs in nonclassical tissues of diabetic animals, we isolated the bone marrow cells, which contain precursors of the main bone-forming cells, such as preosteoblasts and preosteoclasts. RT-qPCR study of isolated bone marrow cells demonstrated that in diabetes mRNA levels of *Cyp27b1* (Figure 4) and *Vdr* (Figure 5) decreased 3.8-fold (*p* = 0.0001) and 9.0-fold (*p* = 0.003), respectively, as compared with the control. These changes were further confirmed by decreased protein levels of CYP27B1 and VDR in the bone marrow cells, the expression of which was visualized using immunofluorescence staining and confocal microscopy. Vitamin D_3_ treatment led to partial or full normalization of the parameters studied.

## 4. Discussion

Type 1 diabetes is an autoimmune endocrine disease associated with *β*-cell destruction that leads to insulin insufficiency and chronic hyperglycemia [14]. In recent years, a number of mechanisms for islet autoimmunity have been proposed, one of which is the potential negative impact of vitamin D deficiency. The latest advances in the area of vitamin D biology have enabled us to better understand how vitamin D deficiency can be involved in T1D and its complications [10]. It was shown that calciferols have immunomodulatory effects that directly affect immune cell maturation and cytokine synthesis. On the one hand, a lack of vitamin D can contribute to the development of T1D, since cholecalciferol does not provide an adequate immunomodulatory effect. On the other hand, among the numerous complications of diabetes, the functions of the kidneys, liver, and many other tissues, which normally participate in vitamin D metabolism, are disrupted. Our study was performed to characterize the expression of key molecules involved in the metabolism and signaling of vitamin D in different tissues in experimental T1D and to assess the efficacy of cholecalciferol to correct the changes associated with diabetes.

Vitamin D metabolism is carried out in various tissues and organs, among which the main role is assigned to the liver and kidneys. A significantly smaller endocrine role, but much more considerable in para/autocrine regulation of cellular functions, is played by extrarenal tissues [15]. The first step of vitamin D hydroxylation occurs in the liver with the help of cytochromes P450, CYP27A1 and CYP2R1 (vitamin D 25-hydroxylases). Our study demonstrated that mRNA levels of both hydroxylases diminished significantly in T1D. Vitamin D_3_ markedly increased the expression of Cyp2r1 without affecting Cyp27a1. Among the factors potentially contributing to the selective effect of cholecalciferol on only one of the two isoforms of hydroxylases, we should take into account the important differences in the physiological functions that these isoforms fulfill in hepatocytes. CYP2R1 is known to be an inducible enzyme with a greater affinity for vitamin D and higher hydroxylase activity than CYP27A1 [16]. It can be assumed that CYP2R1, as an inducible enzyme, is able to respond promptly to cholecalciferol load in diabetes to maintain a sufficient level of 25OHD prohormone for the realization of its physiological functions.

Subsequently, we investigated the expression of hepatic CYP27B1 and VDR, which are responsible for the synthesis of calcitriol and its cellular signaling, that ensures the implementation of vitamin D function locally in the liver [17]. Our results demonstrated a decrease in *Cyp27b1* mRNA, which was accompanied by the downregulation of its protein level. We observed similar changes with respect to the expression of VDR in diabetic liver. These findings can be supported by scientific data on the relationship between vitamin D insufficiency and declined VDR expression in hepatic steatosis [18]. Decreased expression of CYP27B1 and VDR, which we established, is most likely a consequence of vitamin D deficiency and inadequate production of the 25OHD prohormone in the liver of diabetic animals. Cholecalciferol treatment significantly attenuated the harmful effect of diabetes on CYP27B1 and VDR expression in hepatic tissue, which additionally supports the association of these changes with the reduced vitamin D bioavailability.

It is known that *Cyp27b1* gene expression in the kidneys is regulated by a number of physiologically important substances, such as circulating Ca^2+^ and inorganic phosphorus, parathyroid hormone, fibroblast growth factor-23, and, directly, by calcitriol [2, 8]. According to the available data, vitamin D status negatively correlates with CYP27B1 synthesis [19]. Consistent with this, we found that a decrease in 25OHD blood level in experimental animals led, most likely, to a compensatory increase in the synthesis of both CYP27B1 mRNA and protein, and vitamin D_3_ administration reversed these parameters to control values. The positive effect of cholecalciferol is possibly achieved via 1,25(OH)_2_D_3_ interaction with VDR, which binds to the promoter region in the *Cyp27b1* gene and controls its expression. We found that, together with the decreased availability of vitamin D in diabetics, there is a compensatory elevation of renal VDR synthesis. This mechanism can be explained by the assumption that at low concentrations of 25OHD, an increase in 1*α*-hydroxylase in the kidneys ensures the conversion of calcidiol at the highest possible level.

Current evidence suggests that 25OHD directly affects bone mineralization as the skeleton can be referred to a site of 1,25(OH)_2_D extrarenal synthesis [20]. Bone tissue VDRs are known to be predominantly localized on osteoblasts, while 25OH-1*α*-hydroxylase can be expressed both in osteoblasts and osteoclasts. Our results revealed a diabetes-evoked decrease in bone tissue levels of VDR mRNA and protein that can reflect the impairment of osteoblast-mediated osteosynthesis. The reverse effect was observed on *Cyp27b1* gene transcription, which, as we have shown, increased significantly in diabetics. WB indicated that transcriptional activation of *Cyp27b1* gene expression was not sufficient for further protein synthesis of the 25OH-1*α*-hydroxylase, since the CYP27B1 protein level remained unaffected. We can speculate that the difference in the synthesis of CYP27B1 mRNA and protein may indicate its distinctive expression in different types of bone tissue cells, in particular its probable blockage in osteoblasts and, conversely, activation in osteoclasts. Notably, CYP27B1 expression in osteoclasts increases in direct proportion to the degree of their differentiation and activation that usually correlates with increased bone resorption [20]. The imbalance of VDRs and CYP27B1 in bone tissue from diabetic rats found in this study may reflect the changed ratio of osteoblasts to osteoclasts with a shift to the formation of active osteoclasts, which can cause bone resorption and lead to the development of secondary osteoporosis. Vitamin D_3_ treatment can help normalize molecular and cellular mechanisms in the coupling of bone formation to resorption, which results in restoration of osteoblast/osteoclast balance and improve bone remodeling.

Osteogenesis is a highly regulated process, in which subpopulations of bone marrow cells differentiate into mature skeletal tissues to maintain and repair the skeletons [21]. Precursors of basic bone-forming cells (osteocytes and osteoblasts) are mesenchymal stem cells localized in the bone marrow. Osteoclasts are derived from precursors of the myeloid/monocyte lineage that circulate in the blood after their formation in the bone marrow [22]. A number of research efforts indicate that changes in the differentiation and functional activity of osteoblasts play a decisive role in the occurrence of secondary osteoporosis in T1D due to hyperglycemia, generalized oxidative stress, and inflammation [23]. High glucose level induces the synthesis of proinflammatory cytokines that leads to a delay in the maturation of osteoblasts. In addition, hyperglycemia increases PPAR*γ* synthesis by liver cells, which reverses the differentiation of macrophages from osteoclasts to adipocytes [24]. In keeping with the above, we established reduced levels of *Vdr* mRNA in the bone marrow cells isolated from the diabetic rats as well as decreased VDR immunofluorescence. These results may indicate a slowdown in the differentiation of bone marrow precursors, which can lead to diabetes-associated defects in osteosynthesis. Cholecalciferol was effective in correcting the revealed disturbances that confirms the possible role of vitamin D deficiency and impairments of its metabolism in the development of bone marrow cell dysfunction related to diabetes.

## 5. Conclusion

Collectively, our data indicate that T1D development causes vitamin D deficiency and associated vitamin D-endo/para/autocrine system disruption in “classical” organs and extrarenal tissues involved in vitamin D metabolism and 1,25(OH)_2_D signaling. With a general decrease in the synthesis of CYP27B1 and VDR in extrarenal tissues, a compensatory elevation of their expression in the kidneys is most likely. Cholecalciferol administration as a potential hydroxylation substrate and precursor of vitamin D-hormone partially or completely attenuated diabetes-induced abnormalities in the vitamin D-endo/para/autocrine system. Maintaining a sufficient level of vitamin D in diabetes contributes to the normalization of impairments in the metabolism of vitamin D and VDR-mediated cellular signaling.

## Figures and Tables

**Table 1:** Glucose and 25OHD levels at 6-week postinitiation of diabetes and after vitamin D_3_ treatment.

	Whole blood glucose level, mmol/L	Blood serum 25OHD level, nmol/L
Control	4.9 ± 0.2	97.5 ± 5.2
Diabetes	26.8 ± 2.9^∗^	50.2 ± 3.0^∗^
Diabetes + 100 IU vitamin D_3_	21.7 ± 2.5	71.0 ± 3.1^#^

Results are expressed as mean ± SEM. ^∗^*p* < 0.05 versus control; ^#^*p* < 0.05 versus diabetes, *n* = 6.
